# Enzymatic complexes across scales

**DOI:** 10.1042/EBC20180008

**Published:** 2018-10-12

**Authors:** Panagiotis L. Kastritis, Anne-Claude Gavin

**Affiliations:** 1 Interdisciplinary Research Center HALOmem, Charles Tanford Protein Center, Martin Luther University Halle-Wittenberg, Kurt-Mothes-Straße 3a, Halle (Saale) 06120, Germany; 2 Institute of Biochemistry and Biotechnology, Martin-Luther University Halle-Wittenberg, Kurt-Mothes-Straße 3, Halle (Saale) 06120, Germany; 3 Structural and Computational Biology Unit, European Molecular Biology Laboratory (EMBL), Heidelberg 69117, Germany

**Keywords:** enzymology, electron microscopy, glycolysis, protein-protein interactions

## Abstract

An unprecedented opportunity to integrate ~100 years of meticulous *in vitro* biomolecular research is currently provided in the light of recent advances in methods to visualize closer-to-native architectures of biomolecular machines, and metabolic enzymes in particular. Traditional views of enzymes, namely biomolecular machines, only partially explain their role, organization and kinetics in the cellular milieu. Enzymes self- or hetero-associate, form fibers, may bind to membranes or cytoskeletal elements, have regulatory roles, associate into higher order assemblies (metabolons) or even actively participate in phase-separated membraneless organelles, and all the above in a transient, temporal and spatial manner in response to environmental changes or structural/functional changes of their assemblies. Here, we focus on traditional and emerging concepts in cellular biochemistry and discuss new opportunities in bridging structural, molecular and cellular analyses for metabolic pathways, accumulated over the years, highlighting functional aspects of enzymatic complexes discussed across different levels of spatial resolution.

## An unprecedented opportunity for enzymology to bridge its own scales

**Figure 1:**
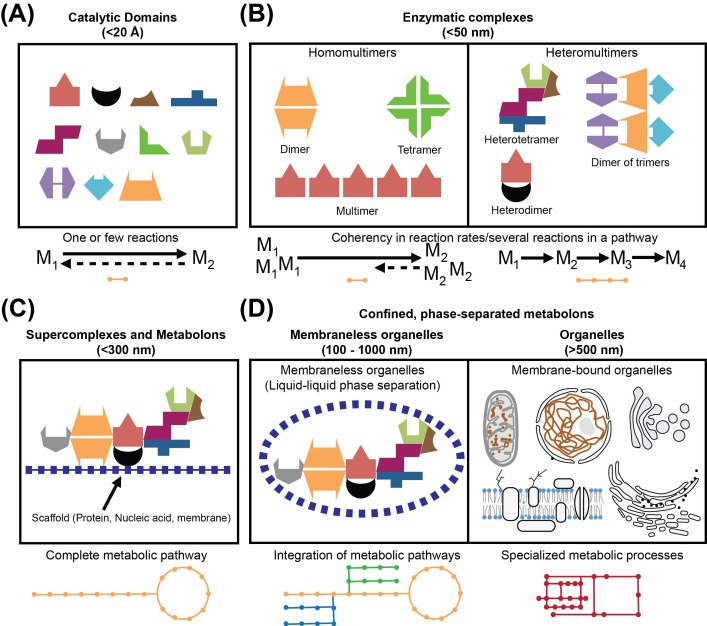
Levels of metabolic organization (**A**) Catalytic domains accelerate one or few biochemical reactions; (**B**) enzymatic complexes may form homomultimers or heteromultimers, by organizing enzymatic domains to efficiently perform more complicated catalytic reactions. For example, by homomultimerization of enzymatic domains, enzyme complexes achieve efficient catalysis of higher concentrations of metabolites. Heteromultimerization may co-localize sequential metabolic enzymes of parts of metabolic pathways. (**C**) A complete metabolic pathway may be organized on scaffold molecules and its participating enzymes transiently interact to form an assembly of its individual components, therefore achieving regulation of the metabolic pathway as function of cellular needs. (**D**) Enzymatic complexes and metabolons may be confined in membrane-less or membrane-bound organelles, therefore, further increasing catalytic efficiency or localizing specialized metabolic processes (see text).

Current methodological leaps in biochemical research have not only provided unprecedented evidence regarding the higher order organization of biomolecular structures in the cellular context [1], or enzyme higher order assemblies in cellular extracts [2], but also provided insights into how cellular context may affect the organization of metabolic pathways [3]. Observations of cellular and molecular organization are inherently associated with the level of resolution that we are able to attain: for understanding those, it is important to analyze the inherent connectivity between elements of metabolic pathways and their role in cellular function. This means that on the basic level, cellular biochemistry is composed of catalytic domains that compose the fundamental units of enzymes (Figure 1A). Enzymes may oligomerize, form homomultimers, heteromultimers (Figure 1B) and higher order supramolecular complexes and transiently interact with consecutive protein complexes (Figure 1C). Enzymes are also tethered to cellular structures, for example membranes, the cytoskeleton or protein/RNA scaffolds, dynamically compartmentalize into membrane-bound or protein-based organelles or even transient microcompartments (Figure 1D), which coherently assemble and function to ultimately orchestrate cellular metabolism with unprecedented precision. The interconnectivity of these different levels of metabolic complexity and how these affect cellular function in the light of current advances in enzymological research are the focusses of this review.

## Catalytic domains: the basic units of metabolic pathways

The basic units of metabolic pathways are the series of domains that catalyze the complex chemistry of life. Most biochemical reactions may also occur in the absence of catalytic domains and non-enzymatic reactions have widespread roles in cellular metabolism [4]. However, catalytic domains are fundamental because they ensure that kinetics and thermodynamics of chemical reactions are tunable and compatible with life. This explains their ubiquitous occurrence in living systems. For example, a simple search in the database of biological structures (PDB, www.pdb.org/) would reveal that over 50% of its current entries (72859 out of 140109 entries) correspond to a biomolecule with enzymatic function (Table 1). Compared with spontaneous reactions, those catalyzed by domains: (i) are considerably faster (in the order of 1000000 to 1000000000 fold), (ii) occur under physiological conditions (physiological T and pH), (iii) tend to be more specific, (iv) can be regulated (via post-translational, allosteric modification of the catalytic domains), and (v) are subject to evolutionary plasticity. Decades of enzymological research already contributed to the biochemical and/or structural characterization of thousands of catalytic domains (e.g. 7288 non-redundant in the BRENDA database, http://brenda-enzymes.org/). This led to the deciphering of some of the principles responsible for the mechanistic coupling between the 3D structure of enzymes and their catalytic activity, and to the current classification scheme of enzymes (e.g. EC 1.X.X.X - 6.X.X.X, Table 1). As a side note, enzymes are very attractive for biotechnological purposes, because they vastly differ from ordinary chemical catalysts.

**Table 1:** Classification of enzymatic reactions and related information

Reaction Type	Schematic	Enzymatic class	Number of (redundant) structures	Number of unique reactions
Redox reactions	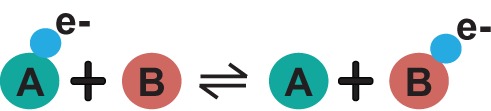	Oxidoreductases (EC 1.)	12581	2143
Single replacement/transfer	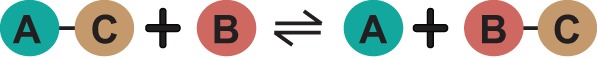	Transferases (EC 2.)	23020	2045
Double replacement/transfer (acid-base reactions)	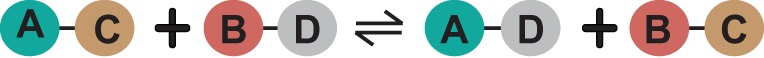	Hydrolases (EC 3.)	26942	1797
Decomposition (bond breaking)	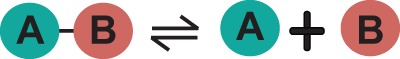	Lyases (EC 4.)	5307	771
Isomerization (bond rearrangement)	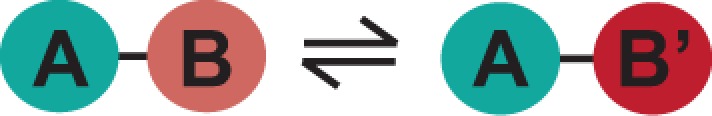	Isomerases (EC 5.)	2985	306
Synthesis (bond formation)	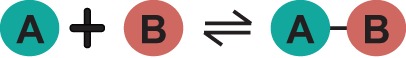	Ligases (EC 6.)	2024	228

Usually, enzymatic reactions are interconnected, and each catalytic domain performs one (or a few) catalytic step(s) in highly complex metabolic pathways. A prominent example, are the ten enzymes of the glycolytic pathway, which break down sugar in our diet and transform it to pyruvate. Glycolysis is at the center of cellular metabolism, and contributes to energy production (ATP) and anabolism (e.g. the pentose phosphate, one carbon pool). Current databases, such as KEGG or BRENDA, provide useful, comprehensive resource describing the metabolic routes followed by nutrients and metabolites, and the physical and chemical properties connected to the catalytic functions (Table 2). Without questioning the practicality of these charts, one cannot help feeling that they only provide a blue print, and generally, the spatial and temporal organization of the pathways *in vivo* remains elusive.

**Table 2:** Online databases including enzymatic information and pathway data

Database	Link	Scope
**General information on enzymes**		
BRENDA	https://www.brenda-enzymes.org/	Molecular and biochemical information on enzymes
KEGG	https://www.genome.jp/kegg/	Computational representation of pathways
MetaCyc	https://metacyc.org/	Metabolic pathways and enzymes
EC2PDB	https://www.ebi.ac.uk/thornton-srv/databases/enzymes/	Known enzyme structures that have been deposited in the Protein Data Bank (PDB)
FunTree	https://www.ebi.ac.uk/thornton-srv/databases/FunTree/	Evolution of enzyme function within domain super families
**Feature-specific databases**		
Recon3D/VMH	http://vmh.life	Human metabolic network reconstruction with pharmacogenomic associations, phenotypic data, and structural information for proteins and metabolites
STRENDA	https://www.beilstein-strenda-db.org/strenda/	Repository of functional enzymology data
SABIO-RK	sabio.h-its.org	Information about biochemical reactions and kinetic rate equations
ASD (allosteric database)	http://mdl.shsmu.edu.cn/ASD/	Resource for structure, function, disease, and related annotation for allosteric macromolecules and modulators
MACiE	https://www.ebi.ac.uk/thornton-srv/databases/MACiE/	Database for enzyme reaction mechanisms
eQuilibrator	http://equilibrator.weizmann.ac.il/	Thermodynamics calculator for biochemical reactions
ORENZA	http://www.orenza.u-psud.fr/	Enzyme activities for which no sequences are available in the main sequence protein databases
**Nomenclature databases**		
IUBMB	https://iubmb.org/	Assignment of EC numbers
ExplorEnz	https://www.enzyme-database.org/	Interface for enzyme nomenclature
IntEnz	https://www.ebi.ac.uk/intenz/	Contains recommendations of IUBMB on the classification of enzyme-catalyzed reactions
ENZYME	https://enzyme.expasy.org	Information relative to the nomenclature of enzymes
**Databases of enzyme classes**		
MEROPS	https://www.ebi.ac.uk/merops/	Database for proteinases
REBASE	http://rebase.neb.com/rebase/	Database of information about restriction enzymes and DNA methyltransferases
CAZy	http://www.cazy.org/	Classification of enzymes involved in the synthesis, metabolism, and transport of carbohydrates
PeroxiBase	peroxibase.toulouse.inra.fr/	Centralization and annotation of most of the peroxidase superfamilies
ESTHER	http://bioweb.ensam.inra.fr/ESTHER/general	Database for α/β-hydrolases
HCS	www.enzyme.chem.msu.ru/hcs/	Hierarchical classification of catalytic sites of hydrolases
**PTM enzyme database**		
PTP	http://ptp.cshl.edu/	Database for protein tyrosine phosphatases
EKPD	http://ekpd.biocuckoo.org/	Kinase and phosphatase database
KinBase	kinase.com/web/current/kinbase/	Includes kinomes, classification, disease association, and an extensive database of protein kinase genes

## Organization of metabolism – molecular scaffolds and protein complexes

Catalytic domains do not operate in isolation, but instead frequently form molecular machines or protein complexes that integrate enzymatic activities (Figure 1). Sometimes, several catalytic sites – that catalyze subsequent steps in a metabolic process – are present on the same polypeptide chain (e.g. fatty acid synthase (FAS)). In other cases, the assembly of a molecular machine implies direct domain–domain interactions and/or the presence of molecular scaffolds (nucleic acids, membranes, cytoskeleton etc). These principles are not mutually exclusive, and represent a level of modularity that allow adaptation to local needs, and the acquisition of new function.

## New properties emerging from homomultimerization

The chemical process that converts monomers into macromolecular complexes through a finite degree of polymerization is termed as *homomultimerization*. Most enzymes form higher order states of their own functional units via *homomultimerization in vitro*, and depending on the concentration and physical-chemical properties of the solvent, they can adopt diverse oligomerization states, ranging from monomers, dimers, larger assemblies and even fibers, but their physiological relevance and functions *in vivo* remain, generally, elusive. It is possible that the process contributes to the fine-tuning of the binding affinities for the substrates, thus facilitating the association events and further stabilizing the enzyme structure.

Another important aspect is the availability of metabolites inside the cell. Indeed many are not randomly distributed, but form highly localized gradients, e.g. that of protons which ATP synthase uses to power synthesis of ATP [5], the gradients of lactate observed in the central nervous system [6], or the gradients of glycolytic ATP induced during cell mobility [7]. Therefore, an oligomeric state of an enzyme may take advantage of local concentrations of substrates, decreasing fruitless associations by burying molecular surface areas that are unimportant for the interaction, while at the same time increasing the surface area covered by active sites.

A particularly well-studied example is the unfoldase p97, a prominent target for cancer drug development [8]. p97 harnesses energy from ATP hydrolysis to extract substrates from various cellular structures. It forms a homohexameric ring-like structure composed of 12 catalytic domains, and, interestingly, half of those are primarily required for oligomerization, while the rest play a major role in ATP hydrolysis [9,10]. Recent high-resolution cryo-EM structures [11] suggest that the binding of ATP induces conformational changes, which lead to a 7 Å contraction of the hexameric ring. In this case, homomultimerization triggers the emergence of a new molecular function – ATP-induced contraction – that can be exploited pharmacologically with the development of allosteric inhibitors [12,13].

Enzymes have been frequently found to form fibrils *in vivo* especially after cellular stresses such as starvation [14,15]. Besides their roles in stress response, fibers of metabolic enzymes serve a multitude of cellular function (besides metabolism), and for example, work as scaffold for the organization of biological processes [16]. The notion that fibers of metabolic enzymes sometimes exert new, unexpected functions depending on their oligomerization state motivates recent efforts to resolve their 3D structures using a variety of EM methods [17–23].

Sickle-cell hemoglobin is a prime example of pathological fibers associated with enzyme function [24]. Red blood cells that contain mainly sickle hemoglobin (HbS) lose their ability to keep the round, soft, spongy shape and texture of a normal red blood cell. The HbS sticks together inside the red blood cells and forms long stiff rods, causing it to become hard and rigid and to assume the shape of a farmer’s sickle. Proof that nucleation of polymers of mutant hemoglobin are the cause of the disease come from knockout sickle-cell mice, which were cured by delaying HbS polymerization [25].

## Heteromultimerization provides advantages to metabolic regulation

**Figure 2:**
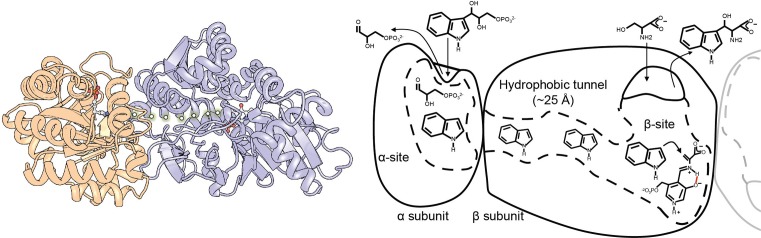
Substrate channeling in Tryptophan synthase On the left, a cartoon representation of two catalytic domains of Tryptophan synthase; small molecules are also shown bound to the catalytic sites, which communicate via a hydrophobic tunnel shown with yellow dots. On the right, the mechanism of substrate channeling in Tryptophan synthase is shown from the α to the β subunit. Note that the molecule is a dimer of dimers (faded part on the right is the continuation of the symmetric dimer) and therefore, mechanistic complication is expected via, e.g. communication of allosteric sites.

A biomolecule containing two or more different polypeptide chains is termed as *heteromultimer*. Heteromultimers, and especially in the form of larger protein complexes, provide a compelling view of interconnected metabolic reactions, which may serve to shuttle intermediates along a particular pathway, especially in cases where substrates are present at concentrations lower than their cognate enzymes. This mechanism, dubbed as substrate channeling, has various advantages over freely diffusing biomolecules [26], including: (i) tighter control of metabolic flux; (ii) protection of reactive and/or toxic intermediates; (iii) increased catalytic efficiency (although catalytic efficiency could also not increase or be advantageous [27]); and (iv) decreased diffusion of intermediates away from the reaction sites. Channels are compartments at the nanometer scale, and facilitate the transfer of the intermediates between allosterically coupled active sites [28]. Their structure, size, and physicochemical nature highly correlates with the nature of the intermediate being shuttled. They can be transient or permanent, hydrophobic in nature [29], hydrophilic [30], electrostatic [31], already present in the structure of the tethered enzymes or be formed after the metabolites are bound [32]. Evidence for such mechanisms can be traced in genes, where two consecutive enzymes are fused in one gene [33]. The classical case of the heterotetrameric (α_2_β_2_) tryptophan synthase, which catalyzes the final two steps in the biosynthesis of tryptophan, exhibits a striking 25 Å hydrophobic tunnel which connects the two reaction centers in which the highly hydrophobic indole moiety is safely shuttled (Figure 2).

**Figure 3:**
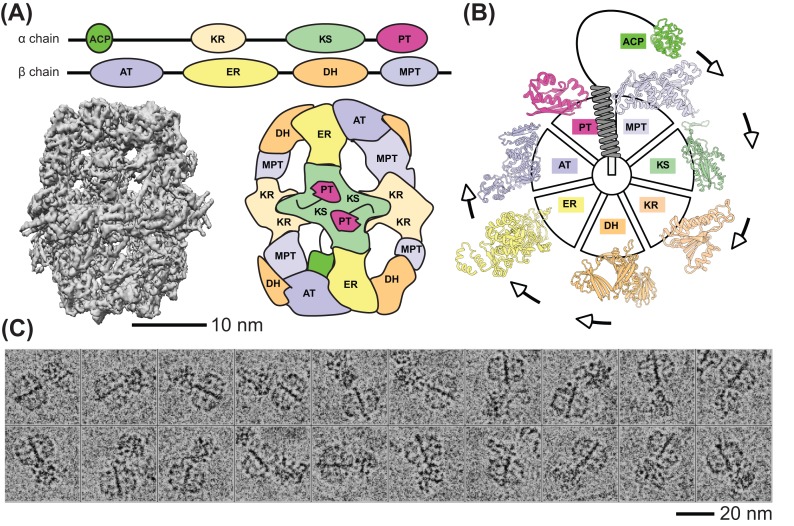
Substrate channeling mechanisms in FAS (**A**) Fungal FAS is composed of two chains, each of which includes four catalytic domains. These domains (explained in the Abbreviations list) are organized in a highly complex 2.6 MDa structure, and each is found six times in the structure, therefore forming 48-catalytic domain biomolecule, highly intricate in the organization of the catalytic domains. (**B**) A simplified mechanism of substrate shuttling in fungal FAS, where the acyl carrier protein (ACP) brings the newly synthesized fatty acid sequentially to each of the catalytic domains of FAS. Note that this wheel-like catalysis is present six times in the protein complex, and therefore, highly intricate allosteric communication is expected, which by now is completely unknown. (**C**) Single-particles from acquired cryo-electron micrographs ([2] and unpublished data) showing the direct interaction between FAS and various other protein complexes, possibly forming a metabolon in fatty acid metabolism. The biomolecules often show connecting densities, clearly illustrating the interconnectivity of large protein complex assemblies and their direct interaction.

These heteromultimers can even form higher, highly complex molecular weight assemblies, for example reflecting a complete metabolic pathway [34] (FAS, Figure 3). Such an adaptation integrates different metabolic routes and contributes to the overall metabolic coherence. A striking form of interconnected metabolic centers exists in FAS, an enzymatic complex in which all steps of the synthesis of fatty acids occur. Interestingly, all active sites are found in the protein complex structure, expressed in two multi-enzyme polypeptide chains that assemble in a gigantic 2.6-MDa complex in an α_6_β_6_ stoichiometry in lower eukaryotes (Figure 3A), but in an a_2_ homodimer in higher eukaryotes. Various high-resolution structures of FAS from yeast [35], thermophilic fungi [2,36], and higher eukaryotes [37] have provided insights into its higher order molecular architecture [38]. An acyl carrier protein is held by a disordered region, which shuttles sequentially the substrate from one enzyme to the next, in a reaction dubbed as ‘covalent channeling’ (Figure 3B). What is truly impressive in this enzymatic machine is that it includes not one, but six complete catalytic chambers, with each being capable of the full fatty acid biosynthesis pathway. In total, FAS has to regulate 48 redundant enzymatic domains. The mechanism of shuttling is highly intricate and electrostatic mini pathways are hypothesized to be implicated in the process [39]. However, the overall allosteric regulation of the enzymatic domains or the catalytic centers of FAS is still largely unresolved [40], and such conformational programming remains to be decoded, not only for FAS, but also for various biosynthetic higher order biomolecular assemblies [41].

## Metabolons: interacting enzymatic complexes and organization by scaffolds

Even more complex, highly transient biomolecular assemblies may exist, called metabolons. These are defined as the metabolic complexes which often assemble into ephemeral structures [42]. Metabolons are co-localized spatiotemporal assemblies of biochemical processes that minimize product leaking by substrate shuttling. Their formation allows passing the intermediary metabolic product from an enzyme directly as substrate into the active site of the consecutive enzyme. Although substrate shuttling is a feature of protein complexes discussed above, a transient mechanistic coupling of individual protein complexes goes beyond simple assembly of individual protein chains with high affinity, as discussed above, such as in the case of FAS. This is because metabolons may form transiently upon cellular needs and connect highly complex metabolic routes. The hypothesis of metabolons is very advanced in plant metabolism research [43,44], and various evidence exist for their presence, e.g. in membranes of chloroplasts (e.g. photosystems [45]). However generally, this level of cellular organization has remained difficult to study, due to the transient and ephemeral nature of the interactions involved. Recently, cryo-EM combined with biocomputational and proteomic approaches offers an attractive way to visualize such assemblies from cellular extracts at higher resolution [2]. FAS was captured interacting with a CoA carboxylase – another megadalton enzymatic complex – possibly integrating catalytic pathways (Figure 3C) [2]. Another example are enzymatic complexes involved in the central carbon metabolism where different enzyme–enzyme interactions contribute to higher order structure of enzymes with distinct cellular function (recently reviewed in [46]). Metabolons are formed because of the need for compartmentalization of biological processes. They go beyond a few interconnected metabolic steps and often imply entire networks and structural and functional integration of different metabolic routes and pathways. Therefore, metabolons may reflect genuine spatial and temporal coherence in cellular metabolism.

In general, metabolons form upon metabolite [47] or cofactor availability [48]. They assemble around molecular scaffolds [49], such as structural proteins [50] or RNA [51,52]. In other cases (the purinosome [53] or the dhurrin [54] and the glycolytic metabolons [47,55]), they localize at the periphery of various cellular membranes [56] (e.g. the plasma [49], the peroxisomal [50], the endoplasmic reticulum [51], and the mitochondria (e.g. enzymes or transporters involved in the Krebs cycle, and the respiratory chain)). Ions, e.g. Ca^2+^, may also regulate their efficiency (e.g. for the Krebs cycle [57]).

**Figure 4:**
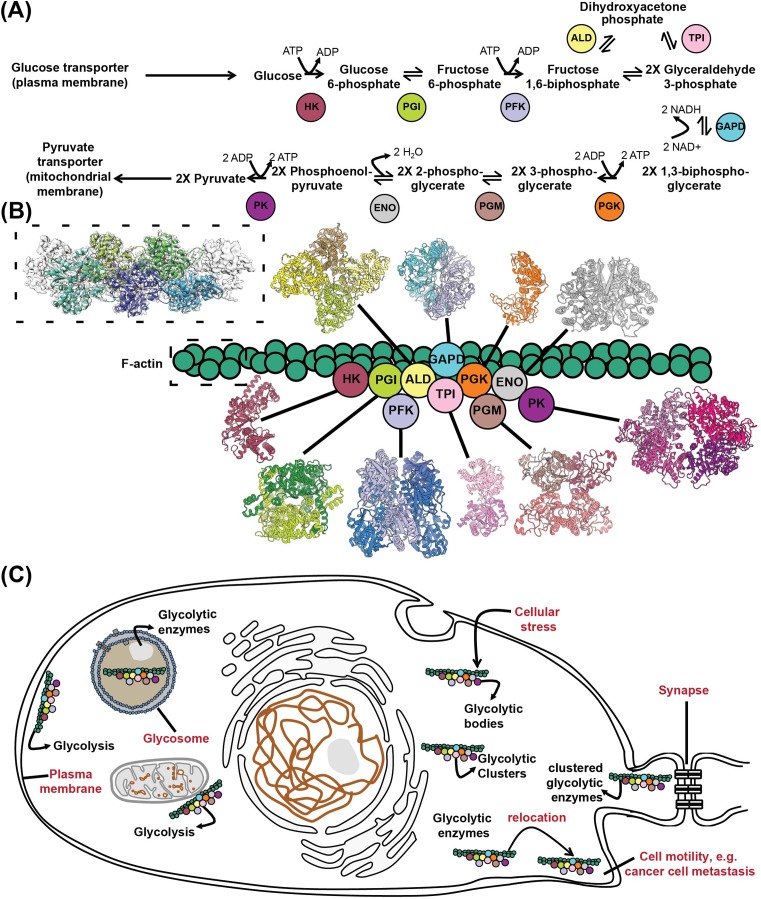
The glycolytic metabolon (**A**) Simplified schematic of glycolysis and acronyms of participating enzymes. (**B**) A hypothesized glycolytic metabolon, where enzymes are interacting, shuttling the intermediates from an active site to another, while enzymatic domains are scaffolded on F-actin. Such a structure represents a classical metabolon which incorporates a full metabolic pathway. (**C**) Localization of the glycolytic metabolon in cells. The glycolytic metabolon has been identified close to plasma membrane of erythrocytes, in specialized organelles in trypanosomes (glycosome), close to mitochondria and on actin or tubulin filaments or formed under cellular stress in yeast. In addition, co-localization of glycolytic enzymes has been identified in HeLa cells, and during e.g. cancer cell metastasis, relocalization has been observed of glycolytic enzymes to aid cell motility. Finally, the glycolytic metabolon has been found to be localized close to synapses in the brain as a cluster of glycolytic enzymes (for details see text).

A particularly interesting example is the long-hypothesized glycolytic metabolon (Figure 4). The glycolytic pathway, shown in Figure 4A, converts one molecule of glucose into two pyruvate molecules in a sequential series of enzymatic reactions catalyzed by ten enzymes in total, of which only three are specific for glycolysis. As the whole pathway, the product of one enzyme serves as the substrate for the consecutive enzyme, it is attractive to assume that these enzymes could directly channel intermediates (Figure 4). Several observations, indeed seem to corroborate this hypothesis. For example, in yeast, interactions of glycolytic enzymes have been identified with large-scale proteomic experiments [58,59]. F-actin provides a scaffold for the assembly of hexokinase (HK), glucose-6-phosphate isomerase (PGI), triose phosphate isomerase (TPI), glyceraldehyde-3-phosphate dehydrogenase (GAPD), phosphoglycerate mutase (PGM), and aldolase (ALD) [50] (Figure 4B). The association of glycolytic enzymes with F-actin increases individual enzymatic activities while protecting against the inhibitory effects of trehalose [50]. Moreover, the activities of several glycolytic enzymes have been found in isolated mitochondria [60] (Figure 4C) and increased glucose flux was associated with compartmentalization of glycolytic enzymes [61]. Under hypoxic conditions, enolase (ENO) forms punctate structures with hexokinase (HK), PGI, phosphofructokinase (PFK), ALD, TPI, GAPD, PGM, and pyruvate kinase (PK) [61] (Figure 4C). Finally, PFK may form cytoplasmic filaments but the functional relevance of the filament structure and its role in metabolon organization remains to be elucidated [18].

Glycolytic enzyme clustering has also been investigated in other systems or organisms. In *Caenorhabditis elegans* neurones (Figure 4C), *Drosophila* flight muscle cells (Figure 4C), and endothelial cells (reviewed in [62]), glycolytic enzymes are compartmentalized, possibly due to the tissue-specific high-energy demand. In *C. elegans*, ALD, GAPD, and PFK, colocalized in clusters at presynaptic sites in neurones under hypoxia or neuronal stimulation (Figure 4C) [49]. Localization of PFK into clusters at presynaptic sites was then hypothesized to be due to energy demands for ATP, and the localization, not necessarily enzymatic activities, of glycolytic enzymes to the presynaptic sites seems to be essential for synaptic function [49]. In *Drosophila* flight muscle (Figure 4C), glycolytic enzymes localize to actin-rich regions of the sarcomere of myofibrils [63], but they missed their co-localization upon glycerol-3-phosphate dehydrogenase (GPDH) knockout [64]. In quiescent endothelial cells, mitochondria and glycolytic enzymes are present uniformly in the cytoplasm. However, relocation of the glycolytic machinery occurs in the lamellopodia and philopodia upon cell migration, indicating, again, that local glycolysis is advantageous in cellular functions requiring high energy [7]. In plant cells, proteomics studies identified glycolytic enzymes interacting with each other [65], and in isolated mitochondria all ten glycolytic enzymes were detected by enzymatic assays [66]. The association of glycolytic enzymes with mitochondria points to the possibility of pyruvate channeling between cytoplasmic glycolysis and mitochondria [47]. The composition of the glycolytic complex is still, however, a mystery. In human erythrocytes, ALD, GAPD, PFK, and PK colocalize to the inner surface of the cell membrane, in an association with the membrane-bound band 3 protein [67].

In mouse erythrocytes, glycolytic enzymes colocalize as well to the membrane with protein band 3, despite lacking the conserved sequences between mouse and human band 3 proteins [68]. The glycolytic metabolon in human erythrocytes interacts with ankyrin (which interestingly has 23 ankyrin repeats, and such helical folds are essential for creating phase-separated membraneless organelles, see below), actin, β-spectrin, and protein band 4.2. Therefore, glycolytic enzymes assemble with other proteins in erythrocytes, forming higher order metabolic supercomplexes [55]. Formation of glycolytic enzymes into complexes in mammalian erythrocytes also depends on the oxygenation state of red blood cells as well as the phosphorylation status of protein band 3 [68,69]. In various cancer cells, formation of a complex in the cytoplasm, termed as the ‘glucosome’, contains both glycolytic and gluconeogenic enzymes [70], and might regulate glucose fluxes. Finally, in protists, glycolysis is confined to well-known organelles, also termed as ‘glycosomes’ [71], which in this case are peroxisomes containing the enzymes responsible for the first six or seven steps of glycolysis in trypanosomatids. To compensate the missing enzymatic activities for glycolysis, trypanosomatids hijack the rest of the glycolytic enzymes from their host organisms to complete glycolysis. Proteomic work has further revealed that, aside from glycolytic enzymes, glycosomes compartmentalize other pathways to co-ordinate metabolism [72]. Without glycosomes, trypanosomatids die in the presence of high amount of glucose, and thus glycosomes appear to be vital in regulating glucose consumption. Overall, the glycosome may provide metabolic plasticity to trypanosmatids for their survival.

Finally, an undiscovered aspect is the role of RNA in assembling such structures. For example, a biochemically isolated glycolytic metabolon was shown to be sensitive to RNase digestion [73], while various enzymes of glycolysis have been found to bind RNA [74]. Therefore, exciting hypotheses have been put forward regarding the scaffolding function of both the mRNAs of those enzymes [51,52], but also long-non coding RNA in organizing glycolysis [75].

## Phase separation with a focus on metabolic complexes and liquid–liquid demixing

Cells, from a thermodynamic point of view, are shockingly ignoring physicochemical theories of condensed matter formation, where high concentration of molecules must generate aggregates. Such a conundrum is exemplified if we consider mitochondria, where enzyme concentrations are the highest as compared with any other organelle, exhibiting values very similar to those of protein crystals. The molecular driving forces governing function in such crowded environments are still elusive, and the mechanisms that the cell employs to avoid spurious biomolecular interactions on the molecular level remain to be elucidated.

Recent work has shown that the cell may contain a suspension of different liquid protein phases [76]. Biomolecular condensates are a class of cellular organelles lacking membrane boundaries that form through phase separation of participating biomolecules. Phase separation has been a recent focus of various reviews [77–82] and even a complete Biochemistry issue has been dedicated to review cellular biology and theoretical physical chemistry of phase-separated processes [83]. Therefore, in the present review the concept is presented with a focus on metabolic complexes and their regulation via the formation of biological condensates by liquid–liquid phase separation.

**Figure 5:**
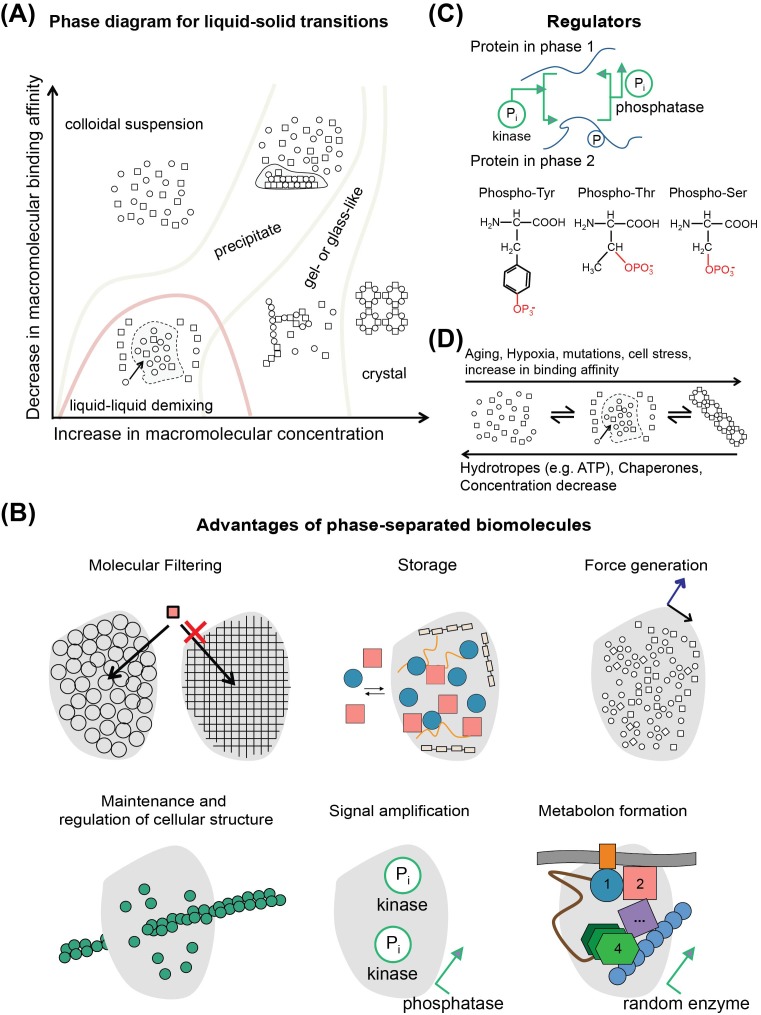
Phase separation and connection to metabolism (**A**) A phase diagram for any biomolecule showing various transitions of its state; a state of liquid–liquid phase separation can be achieved, theoretically for any biomolecule. However, the boundaries of transitions are different for each biomolecule. (**B**) Advantages of phase-separated biomolecules: their unique biophysical properties may regulate mesh size and chemical properties of the condensate, which enables condensates to serve as molecular filters, restricting the diffusion of molecules through their boundaries based on, e.g. their sizes. Phase-separated membraneless organelles can also act as storage deposits, for example of inactive enzymes that would otherwise be waste material (e.g. glyconeogenesis and glycolytic enzymes could in principle phase separate in such granules when one or another competing metabolic pathway is utilized). At the edge of the biological condensate, due to the viscosity and the surface tension of the liquid phase of the condensate, force is generated. Such force could in principle, be utilized by various pathways that might require additional energy to complete. Condensates also function as organizing centers for polymerization, as shown for example, for actin filaments or the microtubule-organizing centers; however, it is yet unknown if enzymatic filaments follow an analogous principle. Signal amplification is also an attractive idea, where for example, inactive metabolic pathways become active after phase-separated signaling molecules, which may reach critical concentrations to regulate the ‘firing’ of the enzymatic pathway. Metabolons may organize on membranes via liquid–liquid demixing and phase-separation processes, having an affinity toward the boundaries of membranes (see also text). (**C**) Illustration of the usual phosphorylation patterns of proteins in phase-separated membraneless organelles which have effect on phase transitions, e.g. by decreasing or increasing biomolecular binding affinity. (**D**) Regulators/effectors of phase transitions.

First, to understand what liquid–liquid phase separation of a biomolecule is, consider the standard phase diagram for liquids (Figure 5A), which is regularly shown in crystallography books. The exact phase diagram will differ for different mixtures of biomolecules, and some mixtures may never achieve some of those states. However, liquid–liquid phase separation has been observed for various processes *in vivo* [76,84–87], and biological condensates have been frequently observed under stress conditions and/or during development, where cellular processes are under strict regulation [77].

Their composition is not entirely known, but RNA and disordered proteins with low complexity regions are ubiquitous components, all reviewed in [77–83]. Several interaction domains have been identified *in vitro* for participating proteins, and interactions via coiled-coils or, in general, helical stacks are promoted. β-strand stacking could be less common, considering the lowest thermodynamic state of the hydrogen-bonding network in β-sheets, but have also been observed in the form of β-zippers. Abundant folds in droplets are RNA recognition motifs that could specifically bind and recognize RNA molecules. How such structures could affect cellular metabolism, and especially enzymatic function is still an unresolved question.

Overall, such biological condensates may serve a multitude of cellular functions, especially considering cellular metabolism (Figure 5B). We can speculate that they may act as: (i) molecular filters because of their unique biophysical properties, (ii) storage deposits, (iii) force initiators by modulating viscosity and surface tension of their drop edges, (iv) organizers of polymers and fibers, (v) signaling mediators via regulating concentrations of signaling molecules, and (vi) promoters of metabolon formation on specific organelles, e.g. on membranes. Importantly, the assembly of enzymes on membranes has been shown for various metabolic pathways, as discussed above, and comprises a specific class of ‘membrane-bound’ or ‘membrane-transport’ metabolons. It would be highly beneficial to investigate whether identified components of metabolons are also found in membraneless organelles that are formed after liquid–liquid demixing.

An important feature of formed biological condensates by liquid–liquid phase separation is their ability to dissolve and organize with astonishing plasticity. This has been shown to happen via chemical modifications of participating biomolecules, especially methylation and phosphorylation (Figure 5C). Astonishingly, it has been shown that ATP could act as a hydrotrope to regulate the assembly of storage granules and liquid condensates [88]. It is also known that during ageing, hypoxia, and stress, cells undergo phase transitions in the cytoplasm toward more dense phases, e.g. forming gel-like granules, solid granules, filaments or solid aggregates, and such phase transitions correlate with disease (Figure 5D). A possible target for reversing such biophysical processes could be molecular chaperones that can act as disassembly factors, as they are also implicated in the homeostasis of functional aggregates *in vivo*. For example, Hsp104 and ClpB collaborate with Hsp70 in eukaryotes or DnaK in bacteria to drive disaggregation processes [12]. Other mechanisms, implying metabolism, can prevent the formation of, dense phases and deleterious protein aggregates, such as the maintenance of small-molecule gradients, e.g. Ca^2+^, ATP or pH gradients or post-translational modifications. Molecular in-cell understanding of the above-mentioned biomolecular recognition principles will greatly facilitate perception of life at the molecular and cellular level.

In summary, metabolism is localized in nano- and micron-sized compartments that coherently assemble biomolecular complexes and that efficiently catalyze, organize, and integrate the complex chemistry of living systems. The notions discussed in this assay are very likely to hold true for other biological processes, e.g. signal transduction, and this idea could be the topic of another review. In the near future, we expect that the combination of emerging methodologies will be critical. For example, advances in cross-linking MS, chemical biology, and cryo-EM may reveal molecular and organization principles in complex specimens (e.g. organs or even entire organisms). Such knowledge will bring us closer to the understanding of the structure of living matter, uncovering structures previously unseen, which may be exploited for biotechnological and medical applications.

## Summary

- The fundamental units of metabolism are catalytic domains that carry most enzymatic reactions required for life. This traditional knowledge of biochemistry only partially explains the role, organization, and kinetics of metabolic enzymes in the cellular milieu.
- Enzymes can oligomerize, form homomultimers, heteromultimers, and higher order supramolecular complexes, and thereby can acquire new, unexpected functions.
- Enzymes are also tethered to cellular structures, for example,membranes, the cytoskeleton or protein/RNA scaffolds, dynamically compartmentalize into membrane-bound or protein-based organelles, or even transient microcompartments, which coherently assemble and function to ultimately orchestrate cellular metabolism with unprecedented precision.

## Abbreviations

ACP: acyl carrier protein
ALD: aldolase
AT: acetyl transferase
DH: 3-hydroxyacyl dehydrase
ER: enoyl reductase
FAS: fatty acid synthase
GAPD: glyceraldehyde-3-phosphate dehydrogenase
HbS: sickle hemoglobin
KR: β-ketoacyl reductase
KS: β-ketoacyl synthase
MPT: manoyl transacylase
PFK: phosphofructokinase
PGI: glucose-6-phosphate isomerase
PK: pyruvate kinase
PT: phosphopantetheinyl transferase
TPI: triose phosphate isomerase
